# Soil quality and ecological benefits assessment of alpine desertified grassland following different ecological restoration measures

**DOI:** 10.3389/fpls.2023.1283457

**Published:** 2023-10-25

**Authors:** Yiran Li, Sijia Hu, Shanxin Lang, Yulin Pu, Shirong Zhang, Ting Li, Xiaoxun Xu, Yongxia Jia, Guiyin Wang, Dagang Yuan, Yun Li

**Affiliations:** ^1^ College of Resource Science, Sichuan Agricultural University, Chengdu, China; ^2^ College of Environmental Science, Sichuan Agricultural University, Chengdu, China

**Keywords:** Zoige Plateau, grassland restoration, minimum data set, soil quality index, ecological restoration effect index

## Abstract

**Introduction:**

Soil quality plays an irreplaceable role in plant growth for restored grassland. However, few studies investigate the comprehensive effects considering soil and vegetation properties during the restoration of desertified grassland, which restrict the virtuous circle of restored grassland ecosystem.

**Methods:**

By setting three restoration patterns of enclosure plus grass (EG), enclosure intercropping shrub-grass (ESG), and enclosure plus sand-barrier and shrub-grass (ESSG) with three different restoration years (≤5, 7–9, and ≥15 years), we selected 28 physicochemical and microbial indicators, and constructed a minimum data set (MDS) to analyze the influences of restoration measurements on soil quality and ecological benefits in alpine desertified grassland.

**Results:**

The results showed that the MDS comprised seven soil quality indicators: silt, total nitrogen (TN), carbon–nitrogen ratio (C/N), total potassium (TK), microbial biomass carbon (MBC), microbial biomass phosphorus (MBP), and fungi. Soil quality index (SQI) and ecological restoration effect index (EREI) in restored grasslands significantly increased by 144.83–561.24% and 87.21–422.12%, respectively, compared with unrestored grassland, and their positive effects increased with extending restoration years. The increasing effects of SQI and EREI were the highest in ESSG, followed by EG and ESG. The increasing rate of SQI began to decrease after 5 years in EG and ESG, while it decreased after 7–9 years in ESSG, and that of EREI in EG was lower than ESSG in each restoration year. Our work revealed that ESSG was the optimum restoration pattern for desertified grassland, and anthropogenic monitoring and management measurements such as applying organic fertilization and mowing return reasonably should be carried out at the beginning of 5 years in EG and ESG as well as 7 years in ESSG to maintain sustainable ecological benefits.

**Discussion:**

The study highlights that soil quality, including microbial properties, is a key factor to evaluate the restoration effects of desertified grassland.

## Highlights

- Microbial properties had vital contributions to the soil quality of alpine grassland.
- Enclosure and sand barriers plus shrub-grass were superior measurements.
- Management practices need to be applied after 5 years in restored grassland.

## 1 Introduction

The grassland ecosystem is one of the most widely distributed terrestrial ecosystems in the world (Cai et al., 2020) and plays a key role in regulating climate change by balancing greenhouse gases (Liu et al., 2020). Grasslands also provide the feed demand of ruminants used for meat and milk production (Liu et al., 2023). Nevertheless, alpine grasslands have been threatened by desertification over the past few decades, leading to decline in water retention, species diversity, and grassland productivity (Liu et al., 2019). Therefore, significant efforts have been made to restore desertified grasslands, such as fencing and reseeding. However, after 7 to 8 years of restoration period, some restored grasslands started to deteriorate once more because there are hardly any management practices based on soil dynamic monitoring and quality assessment. (Dong et al., 2014). Consequently, it is essential to monitor and evaluate the soil quality of restored desertified grasslands in order to increase grassland productivity and retain its varied ecological roles.

The core of scientific soil quality assessment predominantly depends on a reasonable evaluation index system that is differentiated in various environmental conditions (e.g., climate and topography) (Zhou et al., 2020; Mamehpour et al., 2021)—for example, bulk density, organic carbon, and carbon–nitrogen ratio can be used to assess the soil quality of grasslands with temperate maritime climate in Ireland (Askari and Holden, 2014). In total, six soil indicators of total nitrogen, available phosphorus, available potassium, organic matter, salinity, and pH were used to assess the soil quality of grasslands with a temperate continental monsoon climate in the Yellow River Delta, China (Wu et al., 2019). Due to the multicollinearity and redundant information among soil properties, how to screen appropriate indicators was extremely vital for soil quality assessment. Fortunately, minimum data set (MDS) as an effective decision-making tool provides a way to address the issue. This is because the MDS can decrease the data dimension and subjective anthropogenic influence, generating the weights of selected indicators at the same time (Wu et al., 2019), which promote the wide application of MDS in the soil quality assessment of grasslands (Yu et al., 2018; Wu et al., 2019; Zhang et al., 2022). However, the assessment indicators of soil quality only included physicochemical properties and ignored sensitive microbial properties in most of previous researches (Askari and Holden, 2014; Wu et al., 2019), which made it hard to comprehensively elucidate the soil quality and its changes in various grasslands. Therefore, soil microbial properties should be considered in soil quality assessment in restored grasslands.

In a restored grassland ecosystem, ecological benefit is an important concern of restoration effect evaluation (Cai et al., 2020). In general, current research mainly focused on the dynamics of vegetation community characteristics to assess ecological benefits during grassland restoration (Scotton, 2019; Liu et al., 2023). Recent studies reported by Liu et al. (2019) and Hu et al. (2022) found that enclosure increased the vegetation coverage, composition, and biomass. In addition, another experiment adopted the patch dispersal index of shrubland to evaluate the ecological benefits (Wu et al., 2022). It is widely known that favorable soil properties are significant in promoting vegetation growth, contributing to positive pairwise feedback between soil and vegetation (Zhang and Zhao, 2015; Raiesi, 2017). Nevertheless, few research considered ecological benefits assessment indexes that were composed of soil and vegetation properties systematically in degraded grasslands (Liu et al., 2017; Wu et al., 2019; Zhou et al., 2020), which lead to inaccurate results of ecological benefits assessment. Furthermore, Li et al. (2023a) pointed out that more attention should be paid to the dynamics of soil quality combined with ecological benefits to maximize the restoration benefits in desertified grasslands. Therefore, assessing soil quality and ecological benefits requires further investigation in restored grasslands.

The Zoige Plateau, located in the northeastern edge of the Qinghai Tibet Plateau, is an important animal husbandry base and a water conservation area, which is dominated by alpine grasslands, accounting for nearly 50% of the entire plateau (Wang et al., 2014; Yang et al., 2021). Nonetheless, the desertification of alpine grasslands has occurred on account of the combined factors of climate warming, pika damages, and grazing. The grassland area has shrunk by more than 30%, which has affected ecosystem functioning in this area (Liu et al., 2020), mainly manifested as the decrease of biodiversity and the destruction of the water storage function of grasslands (Wu et al., 2015). In response to this increasing trend of desertification, a series of restoration measures such as sowing grass, prohibiting grazing, and setting sand barriers (Hu et al., 2016a; Hu et al., 2016b) has been applied to inhibit the tendency of grassland degradation and restore productivity and ecological function, which have achieved beneficial effects successfully (Wang et al., 2012). Some research indicated that vegetation communities and soil quality have effectively improved through the construction of enclosures and the replantation of grass seeds (Hu et al., 2016a; Liu et al., 2019)—for example, Zhang et al. (2022) reported that long-term ecological engineering enclosure improved the soil quality of alpine desertified grasslands. Similarly, Hu et al. (2022) also found that fencing enclosure promoted vegetation growth as well as soil physicochemical and microbiological properties. However, a lot of scholars mainly paid attention to changes of soil and vegetation properties during the restoration of desertified grassland (Liu et al., 2019; Sun et al., 2020; Wu et al., 2023) and ignored the ecological benefits assessment according to soil quality integrated with vegetation parameters in previous research, making it difficult to comprehensively evaluate the ecological benefits of different restoration measurements. Furthermore, few studies about ecological restoration measurements for desertified grassland simultaneously considered restoration patterns and restoration years. It remains unclear whether scientific artificial management measurements based on soil quality and ecological benefits assessment are required in order to avoid grassland degradation again and maintain the stability of restored grassland ecosystems. Based on different restored grasslands, selecting optimal restoration patterns and exploring the restoration year of applying anthropogenic administration need to be further implemented.

To solve the above-mentioned issues, three types of ecological restoration measurements of desertified grassland, including enclosure plus grass pattern (EG), enclosure intercropping shrub-grass pattern (ESG), and enclosure plus sand-barrier and shrub-grass pattern (ESSG) with restoration years for control group (CK), ≤5 years, 7–9 years, and ≥15 years, were performed in Zoige county on Zoige Plateau, where the problems of grassland desertification were particularly severe since 1990s (Hu et al., 2018b). Therefore, it was hypothesized that (1) microbial properties had vital contribution to grassland soil quality, (2) different restoration measurements show various levels of efficiency on the improvement of grassland soil quality and comprehensive ecological benefits, and (3) each restoration pattern is accompanied by an optimal restoration year cooperating with scientific anthropogenic management measures in restored grassland. To address this hypothesis, the objectives of this study were to (1) construct an appropriate evaluation indicator system for soil quality assessment in alpine grasslands, (2) quantitatively assess the effects of different restoration measurements on soil quality and ecological benefits in desertified grasslands, and (3) explore the optimal restoration year requiring management practices in restored grasslands. This research would promote a virtuous cycle of soil nutrients and vegetation growth in desertified grasslands. Moreover, it can also provide a theoretical basis and practical guidance for the scientific restoration and sustainable management of the alpine grassland ecosystem.

## 2 Materials and methods

### 2.1 Site description

The research area is located in the central and western part of Zoige County on the northeast edge of the Qinghai-Tibet Plateau (33°43′27″–33°51′43″ N, 102°25′40″–102°33′34″ E), China, which is characterized by arid and semi-arid transitions with a cold alpine climate. Annual precipitation fluctuates between 600 and 750 mm (average 656.8 mm), 90% of which occurs from April to October. The annual mean temperature varies in the range of 0.6°C–1.2°C, with monthly averages of −10.8°C in January and 10.9°C in July. The geological landform is a hilly plateau formed by the intense uplift of the Himalayan tectonic movement and neotectonic movement. The elevation varies from 3,400 to 3,450 m above sea level, and the main water systems are the White River and the Black River (tributaries of the Yellow River). It is interspersed with a subalpine region, river valley plains, and several lake depressions. Subalpine meadows, wet meadows, and marshes dominate the main landscape. The soils in this study area were classified as Cambic Coarsic Leptosols in the World Reference Base for Soil Resources (IUSS Working Group WRB, 2022).

Subalpine meadow grasslands have degraded universally by natural and human factors over 20 years ago. The desertified grasslands were widely distributed in Xiaman Town (33°43′27.3″–33°46′13.6″ N, 102°25′35.3″–102°32′45.1″ E), Maixi Town (33°51′35.9″–33°51′43.8″ N, 102°32′56.7″–102°33′34.7″ E), and Axi Town (33°40′59.7″–33°41′4.6″ N, 102°55′57.8″–102°56′4.2″ E) in Zoige County. The degradation characteristics of grasslands were mainly embodied in decrease of vegetation coverage, biomass, and herbage edibleness. To restore degraded grasslands, a series of restoration measures was carried out by Zoige Forestry and Grassland Administration, China, such as planting *Avena sativa*, *Poa pratensis*, *Elymus nutans*, and *Tamarix ramosissima* artificially. Three primary ecological restoration patterns were carried out in each town respectively, including enclosure plus grass pattern (EG), enclosure intercropping shrub-grass pattern (ESG), and enclosure plus sand-barrier and shrub-grass pattern (ESSG). Each pattern has three restoration periods of ≤5 years, 7–9 years, and ≥15 years. We have not adopted anthropogenic management measurements such as mowing return and applying fertilization during grassland restoration.

### 2.2 Plot selection and sampling

**Figure 1:**
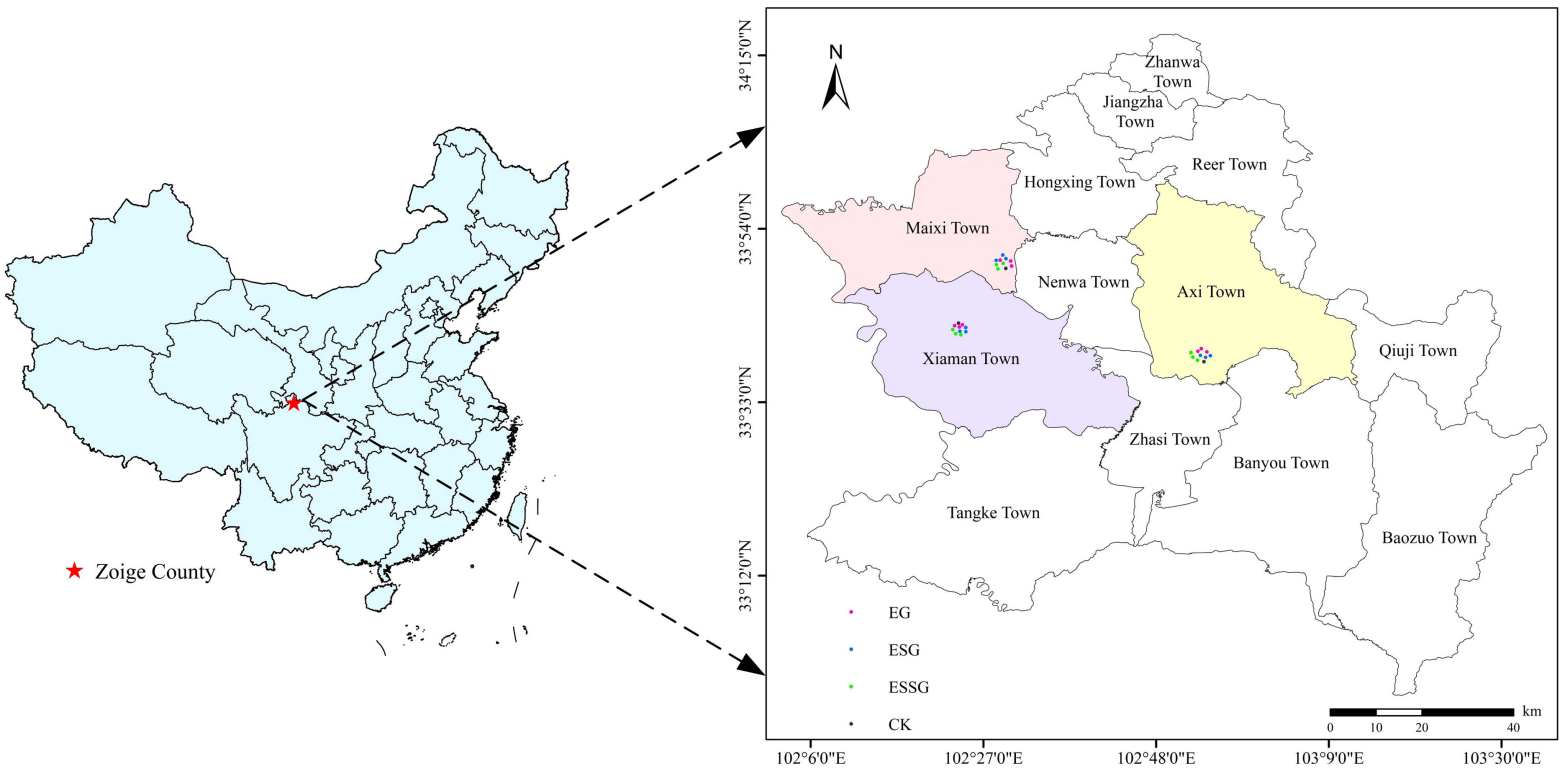
Geographical location of the soil sampling sites in Zoige County.

We set 10 sampling sites, including three restoration patterns with three restoration years, and a control site with unrestored desertified grassland (CK) in each subarea (**Figure 1**). Then, we selected three sampling quadrats (each 2 m × 2 m) in each sampling site based on the theory of biological replicates. The plant community characteristics (e.g., functional groups, density, and coverage) and biomass were investigated, and we obtained plant samples following the method described in Zhang and Zhao (2015) (**Table 1**). The soils (0–10, 10–20, and 20–40 cm) were sampled from five random locations at each quadrat, and 90 samples were collected totally. After removing litter and root material, all soil samples from the same plot were mixed and diminished to 800 g approximately. A part of the soil samples was stored in ice bags and reserved at 4°C for measurement of microbial indicators, and the rest of the soil samples were air-dried to determine the physicochemical indicators. Soil bulk density was measured by the cutting ring method simultaneously.

**Table 1:** Plant community characteristics and biomass in desertified grasslands with different restoration measurements.

Patterns	Restoration years	Vegetation species	Dominant vegetation	Vegetation coverage (%)	Vegetation density (individual m^-2^)	Grass biomass (g m^-2^)	Shrub biomass (g m^-2^)	Shannon–Wiener index
CK	0	*Rosaceae Goosefoot*	*Potentilla chinensis Agriophyllum squarrosum*	0–13	22	2.20	–	1.44
EG	≤5	*Goosefoot*	*Avena sativa Elymus nutans*	53–63	215	97	–	1.69
	7–9			70–77	481	134	–	1.68
	≥15			88–97	661	156	–	1.65
ESG	≤5	*Tamaricaceae Goosefoot*	*Tamarix ramosissima Avena sativa Elymus nutans Poa pratensis*	57–64	171	90	60	1.55
	7–9			63–69	483	135	270	1.52
	≥15			70–85	673	154	448	1.48
ESSG	≤5	*Tamaricaceae Goosefoot Lamiaceae*	*Tamarix ramosissima Poa pratensis Ajuga lupulina*	37–45	101	78	50	1.74
	7–9			45–59	247	102	980	1.59
	≥15			67–75	565	144	4,770	1.25

EG, enclosure plus grass; ESG, enclosure plus shrub-grass; ESSG, enclosure plus sand-barrier and shrub-grass.

### 2.3 Soil sample analysis

Soil particle composition was determined by the simple specific gravity method using Stokes’ law (Lu, 2000). The total organic carbon (TOC) content was determined by the Walkley–Black potassium dichromate sulfuric acid oxidation procedure (Nelson and Sommers, 1983). The permanganate-oxidizable carbon content was determined following the protocol proposed by Culman et al. (2012). The dissolved organic carbon content was determined by using Vario TOC analyzer (Elementar, Germany) after extracting with deionized water (Chen et al., 2003). The TN content was determined by the Kjeldahl method. After 2 mol L^−1^ KCl extraction, ammonium nitrogen (NH_4_^+^) was determined by indophenol blue colorimetry, and nitrate nitrogen (NO_3_^-^) was determined by UV spectrophotometry colorimetry (Lu, 2000). The dissolved organic nitrogen (DON) content was calculated as the difference between TN and TIN (the sum of NO_3_^–^N and NH_4_^+^–N) (Mariano et al., 2016). The total P and available P contents were measured using the colorimetric molybdenum blue method *via* NaOH fusion and 0.5 mol L^−1^ NaHCO_3_ (pH = 8.5) extraction, respectively (Lu, 2000). The total potassium (TK) and available potassium (AK) contents were determined using the flame photometer method after digesting in a nickel crucible with NaOH at 750°C and extracted by 1 mol L^−1^ CH_3_COONH_4_ (pH = 7.0) respectively (Lu, 2000). The number of cultivable bacteria, fungi, and actinomycetes was determined using the dilution plate method (Hou et al., 2014), which were expressed as colony-forming units per gram of soil. The bacteria were incubated in beef protein medium, and the fungi and actinomycetes were cultured in Martin medium and Gauze’s synthetic no. 1 medium (Shamiyeh and Johnson, 1973). After soil sampling was performed using the chloroform fumigation–extraction method, Vario TOC cube (Elementar, Germany) analyzer was used to determine the MBC and microbial biomass nitrogen, and MBP was determined by the colorimetric molybdenum blue method (Brookes et al., 1985; Vance et al., 1987). The urease activity was measured by the phenol-sodium hypochlorite method using urea as substrate, which was expressed as milligrams of NH_4_^+^ per gram of dry soil per hour after incubation at 37°C for 24 h (Samborska et al., 2004). The proteinase activity was determined by the Folin-Ciocalteau reagent and expressed in milligrams of tyrosine per gram of dry soil per hour produced by the incubation of sodium caseinate at 50°C for 2 h (Weber and Tihanyi, 1994). The sucrase activity was measured by the 3,5-dinitrosalicylic acid colorimetric method, expressed as the amount of glucose released per gram per hour of soil sample in 24 h (Frankeberger and Johanson, 1983). The amylase activity was determined by the dinitrosalicylic acid colorimetric method and expressed as the milligram of maltose in 1 g soil after 24 h (Zhang et al., 2011). Both neutral and alkaline phosphatase activities were determined by the disodium 4-nitrophenylphosphate colorimetric method, which were expressed by the release of 4-nitrophenol per gram of soil samples after incubation at 37°C for 1 h from disodium 4-nitrophenylphosphate (Guan, 1986; Tabatabai, 1994).

### 2.4 Soil quality assessment

#### 2.4.1 Minimum data set

The MDS approach was used to establish the index system of soil quality (Andrews et al., 2004), aiming to reduce the indicator redundancy (Jahany and Rezapour, 2020). We collected 28 potential soil indicators in the present work, including physicochemical and microbial properties. During this process, principal component analysis (PCA) was used to group the indicators, and only the components with eigenvalues ≥1 were selected. Then, the indicators with loadings ≥0.5 in the same component were classified into one group. If the loading of an indicator in two or more components was >0.5, the indicator was classified into the group where the indicator had the lowest correlations with other indicators. The norm value intended to screen the assessment indicators, representing the comprehensive loading of an indicator in all components. The norm value of each indicator was calculated by using Eq. (1) as follows (Jin et al., 2021):


(1) $$\text{Norm}i=\sqrt{\sum_{i=1}^{k}\left(uik^{2}\times \lambda k\right)}$$

where Norm*_i_* is the comprehensive loading of indicator *i* in all components with eigenvalues ≥1, *µ_ik_* is the loading of indicator *i* in component *k*, and *λ_k_* is the eigenvalue of component *k*.

The indicator whose norm value was within the 10% range of the maximum value of a group was selected for further correlation analysis (Wu et al., 2019). Subsequently, if the Pearson correlation coefficient of two arbitrary indicators was higher than 0.5, the indicator with a higher norm value was retained in the MDS; otherwise, the indicators were selected.

#### 2.4.2 Soil property scoring

The weight value was calculated by the ratio of norm value for each indicator to the sum of norm values for the selected indicators, as shown in Eq. (2) (Yao et al., 2013). The normalized method is used to convert variables into dimensionless values between 0 and 1 due to the inconsistent units of soil indicators. The relationships among indicators and quality in soils could be divided into three scoring functions: “more is better”, “less is better”, and “optimum is better” (Santos-Francés et al., 2019). The “more is better” function was applied to the selected indicators because of their positive effects on soil quality in this research. Therefore, the degrees of membership for the selected indicators were calculated using the ascending property function, as shown in Eq. (3) (Biswas et al., 2017). After that, the scores for physicochemical and microbial properties were calculated by combining the degree of membership with the weights of indicators for soil samples as in Eq. (4) (Liu et al., 2017).


(2) $$wi=\frac{\text{Norm}i}{\sum_{i=1}^{n}\text{Norm}}$$

where *w_i_* is the weight of each indicator.


(3) $$S_{i}=\frac{X_{\text{max}}-Xi}{X_{\text{max}}-X_{\text{min}}}\;$$

where *S_i_* is the degree of membership of each indicator, *X_i_* is the observed value of each indicator, and *X*_max_ and *X*_min_ is the maximum and minimum value of each indicator, respectively.


(4) $$F_{p}=\sum_{i=1}^{n}\left(wi\times S_{i}\right)$$

where *F_p_* is the score of physical, chemical, and microbial properties, and *n* is the number of soil indicators in the MDS.

#### 2.4.3 Soil quality index

SQI was calculated by the scores of physicochemical and microbial properties in soils and their corresponding weights, reflecting the effects of desertified grassland restoration measurements on soil quality. A larger SQI denotes better soil quality (Liu et al., 2017). The weight values of soil properties were calculated by the commonalities derived from the PCA as shown in Eqs. (5–7) in turn (Mamehpour et al., 2021; Martín-Sanz et al., 2022). SQI was calculated using Eq. (8) (Romaniuk et al., 2011).


(5) apj=fpjλj

(6) $$W_{p0}=\sum_{j=1}^{n}a_{pj}\times E_{j}$$

(7) $$Wp=\frac{W_{p0}}{\sum_{i=1}^{m}W_{p0}}$$

where *a_pj_* is the feature vector of property *p* in component *j*, *f_pj_* is the loading of the property *p* in component *j*, *λ_j_* is the eigenvalue of component *j*, *W_p0_* is the weight value of each property, *E_j_* is the explained variance of component *j*, and *W_p_* is the weight value of property *p* after normalization.


(8) $$\text{SQI}=\sum_{i=1}^{m}\left(Wp\times Fp\right)$$

where SQI is the soil quality index, and *m* is the number of soil properties.

### 2.5 Ecological benefits assessment of restoration measurements

PCA was also used to calculate the weight values of indicators, including vegetation coverage, density, and biomass as well as silt, TN, carbon–nitrogen ratio (C/N), TK, MBC, MBP, and fungi in soils. The calculation method of indicator weights was similar to that of soil property weights in soil quality assessment using Eqs. (1) and (2). The degrees of membership of the above-mentioned indicators were calculated using Eq. (3) because these indicators were in accordance with the function of “more is better” (Mamehpour et al., 2021). The ecological restoration effect index (EREI) of restoration measurements was calculated according to Eq. (4).

### 2.6 Data analysis

The experimental data analyses were performed using SPSS 19.0 (IBM Corp., US) (i.e., maximum, minimum, mean, and standard error). One-way analysis of variance, followed by the Duncan test, was applied to test differences of soil quality indicators among depths, restoration patterns, and years (*p*< 0.05). The correlation coefficients among soil microbial and physicochemical indicators were analyzed *via* Pearson correlation analysis. The figures were drawn by using Origin 2022b (Origin Lab Corp., Northampton, MA, USA).

## 3 Results

### 3.1 Soil quality indicators

The soil quality indicators for unrestored grassland (CK) had no significant difference in three subareas (*p* > 0.05) (**Supplementary Table S1**), and they had no significant differences except bacteria and alkaline phosphatase among depths in the alpine grassland (*p* > 0.05) (**Supplementary Table** **S2**). Accordingly, the weighted average of soil quality indicators from different layers was performed for the same quadrat in this research.

As shown in **Table 2**, the values of soil quality indicators, including physical and chemical properties in restored grasslands, were preferable to those under CK treatment at each restoration year. The soil bulk density and sand content among different ecological restoration patterns were lower than that in unrestored grasslands, and they showed a decreasing trend with increasing restoration years. However, the contents of soil silt, clay, TOC, TN, DON, NH_4_^+^–N, and NO_3_^–^N in restored grasslands were significantly higher than those in unrestored grasslands (*p<* 0.05), and they generally significantly increased with increasing restoration years (*p<* 0.05). Moreover, the contents of soil silt and clay in ESSG were significantly higher than in EG and ESG. Meanwhile, our previous results showed that the soil microbial properties, including microbial abundance, microbial biomass, and enzyme activity, in the three patterns of restored grasslands significantly improved (*p*< 0.05) compared with unrestored grasslands (**Supplementary Table S4**) (Hu et al., 2018a). The improvement effects of the microbial properties enhanced with extended restoration years at each pattern, and those in ESSG were generally better than EG and ESG (**Supplementary Table S4**) (Hu et al., 2018a).

**Table 2:** Effects of restoration measurements on the soil physicochemical properties.

Indicators	CK	EG			ESG			ESSG		
		≤5 years	7–9 years	≥15 years	≤5 years	7–9 years	≥15 years	≤5 years	7–9 years	≥15 years
BD (g cm^-3^)	1.39a	1.17Bb	1.07Ac	0.94Bd	1.23Ab	1.13Abc	1.05Ac	1.25Ab	0.84Ac	0.97ABc
Sand (%)	96.47a	88.94Cb	89.34Ac	89.37Ac	88.03Bb	88.40Ac	88.4Bd	86.37Ab	86.8Bc	86.63Cd
Silt (%)	1.50 d	6.91Ac	6.83Bb	6.83Ba	8.23Ac	7.37Bb	7.53Aa	7.89Bc	7.23Aa	7.20Bb
Clay (%)	2.03b	4.14Aa	3.83Aa	3.8Ba	3.73 Ba	4.23Aa	4.07Ba	5.74Ca	5.97Aa	6.17Aa
TOC (g kg^-1^)	1.24c	3.24Ab	4.67Ab	9.61Aa	3.22Abc	3.78Ab	7.75Ba	2.98Ac	6.99Ab	9.08ABa
TN (g kg^-1^)	0.05d	0.16Ac	0.26Ab	0.42ABa	0.19Ab	0.21Ab	0.38Ba	0.10Bbc	0.23Ab	0.50Aa
C/N	16.84b	20.15Bab	17.95Ab	23.52Aa	17.32Ba	17.38Aa	20.99Aa	26.19Aa	21.42Ab	18.15Ac
DON (mg kg^-1^)	1.30c	1.58Ac	3.22Ab	4.65Aa	2.13Aa	1.41Ab	1.96Bab	1.65Ab	2.94Ab	5.08Aa
NH_4_^+^–N (mg kg^-1^)	0.39d	1.37Ac	2.47Ab	3.75Aa	1.68Ab	1.98Aab	2.67Ba	1.85Ab	2.13Ab	3.40Aa
NO_3_^–^N (mg kg^-1^)	0.46d	1.09Ac	2.11Ab	3.38Aa	1.15Ab	1.55Ab	2.35Aa	1.80Aab	2.38Aab	3.20Aa
TP (g kg^-1^)	0.24d	0.25Bc	0.32Ab	0.35Ca	0.28Bb	0.35Aa	0.39Ba	0.36Ab	0.41Aab	0.50Aa
AP (mg kg^-1^)	1.88c	2.92Ab	2.83Bb	5.00Ba	2.88Abc	4.17ABab	5.47Ba	2.88Ac	4.95Ab	6.57 Aa
TK (mg kg^-1^)	17.96ab	17.76Ab	19.13Aab	19.97Aa	18.44Ab	18.13Bb	17.93Bb	19.30Aa	19.25Aa	18.00Bb
AK (mg kg^-1^)	30.98c	39.20Bab	37.33Bb	48.84Ba	30.26Bb	52.15Aa	50.43Ba	50.13Ab	55.55Ab	68.50Aa

BD, bulk density; TOC, total organic carbon; TN, total nitrogen; C/N, carbon–nitrogen ratio; DON, dissolved organic nitrogen; NH_4_^+^–N, ammonium nitrogen; NO_3_^–^N, nitrate nitrogen; TP, total phosphorus; AP, available phosphorus; TK, total potassium; AK, available potassium.

Different capital letters above each bar indicate significant differences among different restoration patterns at p< 0.05. Different lowercase letters above each bar indicate significant differences among different restoration years at p< 0.05.

### 3.2 Minimum data set of soil quality

**Table 3** indicates that different principal components (PCs) were selected with eigenvalues ≥1 for soil physical, chemical, and microbial properties. PC1 explained 82.39% of the total variance in soil physical properties including four indicators, and soil silt was selected as the appropriate indicator representing physical properties (**Table 3**). In terms of chemical properties encompassing 12 indicators, three PCs explained 79.23% of the total variance, and PC1, PC2, and PC3 explained 59.48%, 11.18%, and 8.57% respectively. Combined with the correlation coefficients of soil chemical indicators, we chose TN, C/N, and TK in the MDS, representing chemical properties on account of their higher loadings and norm values (**Table 3**; **Supplementary Table S3**). Concerning microbial properties involving 12 indicators, two PCs explained 79.42% of the total variance, and PC1 and PC2 explained 71.06% and 8.36% of the variation, respectively. MBC, MBP, and fungi were contained in the MDS because of higher loadings and norm values (**Table 3**; **Supplementary Table S4**). As a result, sensitive and important indicators comprising soil silt, TN, C/N, TK, MBC, MBP, and fungi were selected to establish the MDS of alpine grassland soil quality.

**Table 3:** Principal component loading matrix and norm values of soil quality indicators.

Soil indicators	Physical properties	Chemical properties			Microbial properties		Group	Norm
	PC1	PC1	PC2	PC3	PC1	PC2		
Silt	-0.986						1-1	1.790
BD	0.921						1-1	1.671
Sand	0.869						1-1	1.577
Clay	-0.849						1-1	1.542
TN		0.929	-0.252	0.099			2-1	2.501
NH_4_^+^–N		0.904	-0.109	0.192			2-1	2.426
TOC		0.902	0.145	-0.017			2-1	2.416
AP		0.857	-0.033	-0.247			2-1	2.304
TP		0.856	0.113	-0.128			2-1	2.294
NO_3_^–^N		0.849	-0.085	0.131			2-1	2.275
PXOC		0.813	0.026	-0.061			2-1	2.173
DON		0.769	-0.013	0.308			2-1	2.079
DOC		0.766	0.205	-0.181			2-1	2.069
AK		0.758	0.128	-0.312			2-1	2.054
C/N		-0.156	0.885	-0.345			2-2	1.161
TK		0.129	0.617	0.734			2-3	1.088
MBN					0.948	0.012	3-1	2.768
MBC					0.944	0.039	3-1	2.758
Bacteria					0.932	-0.006	3-1	2.721
Urease					0.926	-0.113	3-1	2.705
Protease					0.912	-0.084	3-1	2.664
Amylase					0.902	-0.097	3-1	2.636
Sucrase					0.881	0.065	3-1	2.574
Neutral phosphatase					0.841	-0.124	3-1	2.459
Alkaline phosphatase					0.822	0.145	3-1	2.403
Actinomyces					0.638	0.383	3-1	1.902
Fungi					0.648	0.533	3-2	1.966
MBP					0.616	-0.707	3-2	1.933
Eigenvalue	3.296	7.137	1.342	1.028	8.527	1.003		
Variance (%)	82.392	59.478	11.183	8.566	71.056	8.360		
Cumulative variance (%)	82.392	59.478	70.661	79.227	71.056	79.416		

PC, principal component; BD, bulk density; TN, total nitrogen; NH_4_^+^–N, ammonium nitrogen; TOC, total organic carbon; AP, available phosphorus; TP, total phosphorus; NO_3_^–^N, nitrate nitrogen; PXOC, permanganate oxidized carbon; DON, dissolved organic nitrogen; DOC, dissolved organic carbon; AK, available potassium; C/N, carbon–nitrogen ratio; TK, total potassium; MBN, microbial biomass nitrogen; MBC, microbial biomass carbon; MBP, microbial biomass phosphorus.

### 3.3 Soil quality

**Figure 2:**
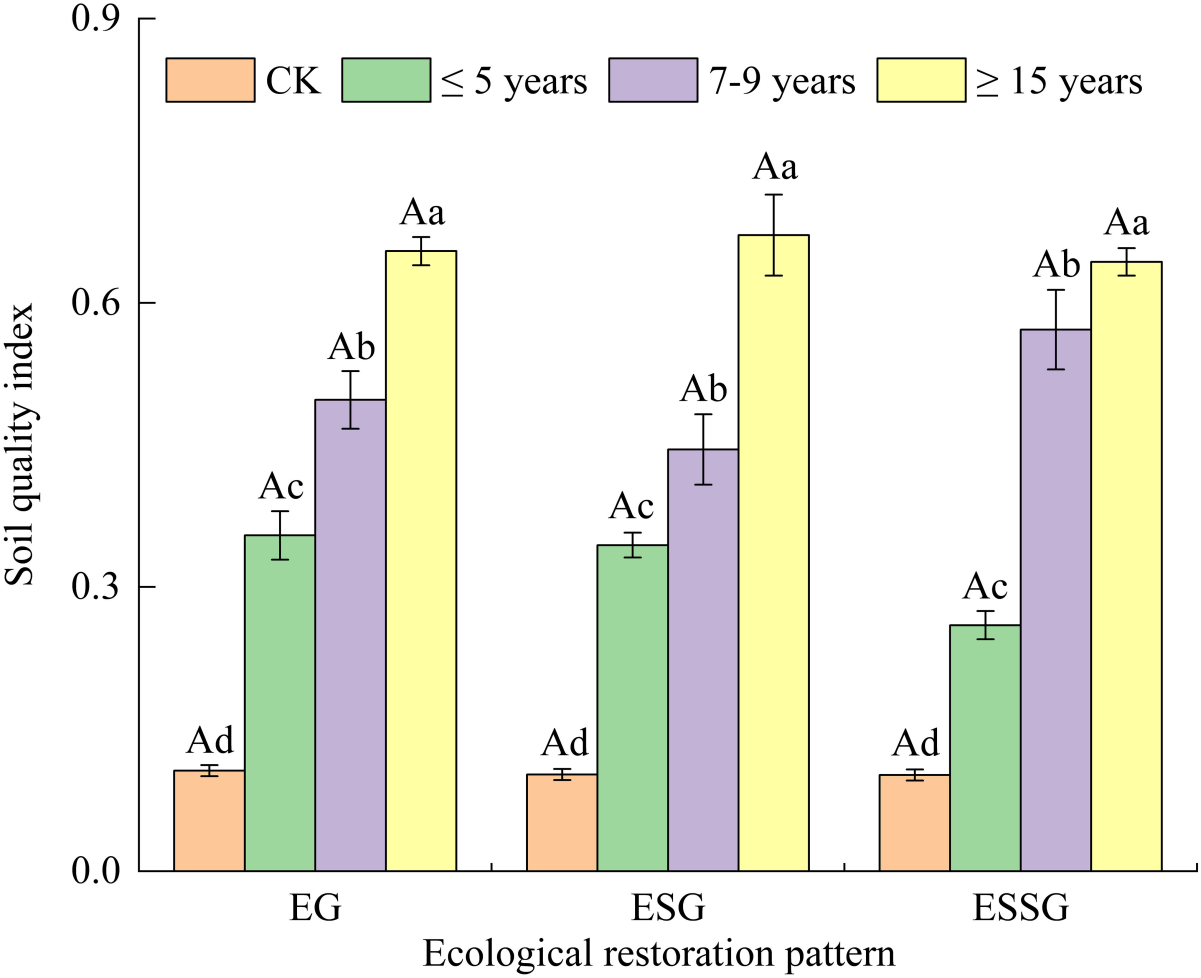
Soil quality index of desertified grasslands with different restoration measures. Different capital letters above each bar indicate significant differences among different restoration patterns at *p*< 0.05. Different lowercase letters above each bar indicate significant differences among different restoration years at *p*< 0.05. Vertical bars denote the standard error of the means. EG, enclosure plus grass; ESG, enclosure intercropping shrub-grass; ESSG, enclosure plus sand-barrier and shrub-grass.

As shown in **Table 4**, the weights of soil physical, chemical, and microbial properties were almost equal. The scores of soil properties increased with increasing restoration years. **Figure 2** shows that the value of SQI varied from 0.102 to 0.671. The SQI in ESSG was higher than EG and ESG overall (**Figure 2**). The SQI in each restoration pattern increased significantly with increasing restoration years. The SQI of ≤5 years, 7–9 years, and ≥15 years increased by 144.83–249.14%, 319.63–463.10%, and 506.28–561.24%, respectively, compared with CK (*p*< 0.05) (**Figure 2**). Moreover, the increasing rate of SQI began to reduce after 5 years in EG and ESG (**Figure 2**), whereas the increasing rate of SQI between ≤5 years and 7–9 years improved by 120.13% compared with ≤5 years in ESSG, which started to decrease after 7–9 years (*p*< 0.05) (**Figure 2**).

**Table 4:** Indicator weights and scores of properties in soils.

Properties	Indicators	Years	Scores of properties		
(Weight, w*_p_*)	(Weight, *w_i_*)		EG	ESG	ESSG
Physical properties	Silt (1.00)	0	0.074		
(0.34)		≤5	0.495	0.399	0.197
		7–9	0.662	0.652	0.795
		≥15	0.722	0.909	0.712
Chemical properties	C/N (0.24)	0	0.079		
(0.34)		≤5	0.057	0.047	0.099
		7–9	0.048	0.049	0.151
		≥15	0.072	0.061	0.049
	TN (0.53)	0	0.009		
		≤5	0.117	0.148	0.059
		7–9	0.211	0.167	0.179
		≥15	0.357	0.320	0.435
	TK (0.23)	0	0.045		
		≤5	0.127	0.152	0.183
		7–9	0.177	0.141	0.181
		≥15	0.203	0.134	0.129
Microbial properties	MBC (0.42)	0	0.030		
(0.32)		≤5	0.109	0.136	0.082
		7–9	0.176	0.161	0.166
		≥15	0.268	0.305	0.319
	MBP (0.28)	0	0.024		
		≤5	0.045	0.046	0.101
		7–9	0.068	0.072	0.125
		≥15	0.139	0.113	0.192
	Fungi (0.30)	0	0.046		
		≤5	0.108	0.101	0.057
		7–9	0.144	0.086	0.108
		≥15	0.200	0.167	0.091

C/N, carbon–nitrogen ratio; TN, total nitrogen; TK, total potassium; MBC, microbial biomass carbon; MBP, microbial biomass phosphorus.

### 3.4 Ecological restoration benefits

**Figure 3:**
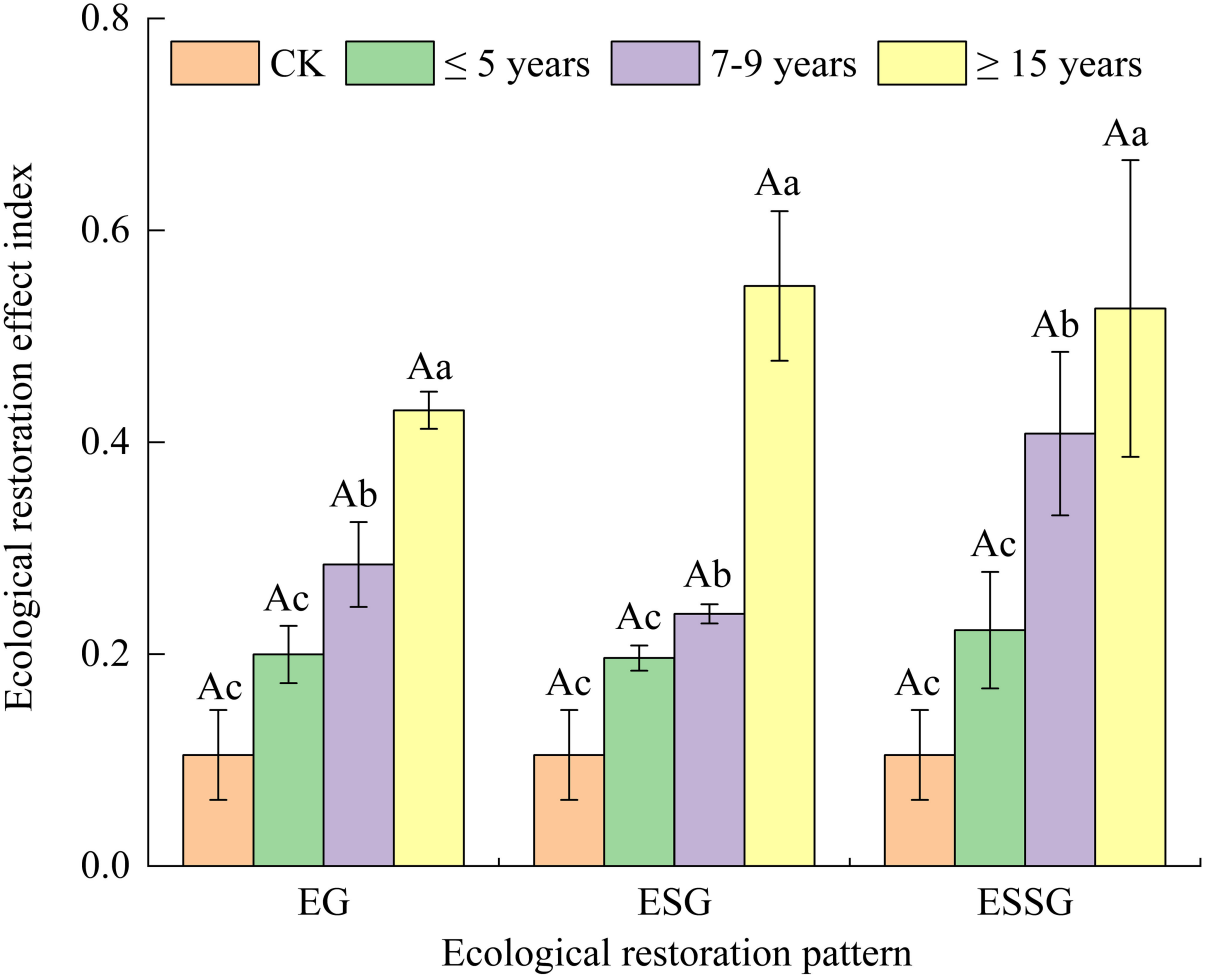
Ecological restoration effect index of desertified grasslands with different restoration measures. Different capital letters above each bar indicate significant differences among different restoration patterns at *p*< 0.05. Different lowercase letters above each bar indicate significant differences among different restoration years at *p*< 0.05. Vertical bars denote the standard error of the means. EG, enclosure plus grass; ESG, enclosure intercropping shrub-grass; ESSG, enclosure plus sand-barrier and shrub-grass.

The value of EREI ranged from 0.104 to 0.547 (**Figure 3**). There was no significant difference among the three restoration patterns (*p* > 0.05), but EREI in ESSG was generally higher than that in EG and ESG, especially before 7–9 years (**Figure 3**). EREI increased constantly with increasing restoration years, of which 7–9 years and ≥15 years significantly increased by 6.93%–107.91% and 93.23%–178.89% respectively, compared with 0–5 years (*p*< 0.05) (**Figure 3**). In addition, the increasing rate of EREI in EG was lower than ESSG. Moreover, the increasing rate of EREI in ESG was lower than ESSG from 5 to 7–9 years, while that in ESG was higher than ESSG after 7–9 years (**Figure 3**).

## 4 Discussion

### 4.1 Evaluation indicator system for soil quality in alpine grasslands

In this research, soil silt, TN, C/N, TK, MBC, and MBP as well as fungi have been selected as soil quality indicators, and they were associated with vegetation growth during grassland restoration. The changes of soil silt content under the application of restoration measurements were the most significant compared with that of sand and clay, similar to the finding of He et al. (2021), which was ascribed to the fact that vegetation restoration could weaken wind erosion, thereby reducing the loss of fine particles (Zhang et al., 2022). The decomposition of plant litterfall increased the soil organic matter under the conditions of planting herbs and/or shrubs plus grazing prohibition in desertified grassland (Yu et al., 2018). Thus, litterfall was enhanced with an increase of aboveground biomass in restored grasslands (**Table 1**), which induced the changes of C/N and TN contents in soils. The potassium (K) requirement for plant growth greatly increased in the middle and late stage of restoration, which could promote the conversion of soil mineral K to AK *via* the mobilization of organic acids and enzymes from K-solubilizing bacteria, and mineral K was the main form of TK in soils (Zhang et al., 2022). The increase of litter input led to the improvement in soil water storage capacity, which was conducive to the reproduction of microorganisms together and further increased the fungi abundance as well as the contents of MBC and MBP in soils (Liu et al., 2017; Zhang et al., 2021). In recent years, some studies have also established the MDS of soil quality assessment based on physicochemical properties in temperate grassland ecosystems (Wang et al., 2021b; Li et al., 2023b). Nevertheless, our results found that the weights of soil microbial properties were almost equal to the soil physicochemical properties and that the soil microbial properties had positive and significant effects on TN (**Supplementary Figure S1**), indicating that microbial properties had non-negligible significance to soil quality in this study area. Therefore, microbial properties should be considered to assess the soil quality of alpine grassland under restoration measurements.

### 4.2 Comparison in the assessment indicators of soil quality among restored desertified grasslands

Different restoration patterns and years had various improvement effects on soil assessment indicators. As far as soil physical properties were concerned, silt content increased, which was similar to the findings of Hu et al. (2022) and can be attributed to the fact that vegetation restoration effectively resisted soil sand outside and promoted the optimization of soil texture (Zhang and Zhao, 2015). Soil structure and texture improved by applying restoration measurements, which may further lead to an increase in soil nutrients (Hu et al., 2022). In this study, the soil nutrient contents of the restored grassland were also significantly higher than those of the unrestored grassland, particularly the significant increase of TN content, similar to the implementation effects of restoration measurements in Shenza County in the Tibetan Plateau as reported by Zhang et al. (2022). This was mainly because the humification of litterfall could directly increase the soil nutrient content, and the improvement of physical properties (e.g., texture, structure, and water retention) might reduce the mineralization of organic matter (Kooch et al., 2022). Previous studies revealed that the vegetation biomass had positive relationships with soil microbial biomass generally (Zhang and Zhao, 2015; Yang et al., 2022). The increase of carbon and nitrogen sources of microorganisms further enhanced the soil microbial biomass (Yu et al., 2018). Therefore, the contents of MBC and MBP as well as the quantity of fungi improved significantly, which was caused by the increase of vegetation biomass together with soil carbon and nitrogen contents in each restoration pattern (**Tables 1**, **2**).

In general, soil quality indicators are gradually optimized due to increased vegetation coverage, density, and biomass (Zhang et al., 2022; Wan et al., 2023). In this research, the vegetation coverage, density, and biomass of restored grassland increased (**Table 1**), thus inducing soil quality indicators gradually ameliorated with the increasing restoration years, which was similar to the findings of Li et al. (2023c). Furthermore, the contents of slit and MBC in ESSG were significantly higher than EG (**Table 2**, **Supplementary Table S4**). These results can be explained by the combined effects of sand barriers and vegetation that were beneficial to sand fixation and water conservation, further effectively improving the soil quality (Wang et al., 2021a; Hu et al., 2022).

### 4.3 Effects of desertified grassland with different restoration measures on soil quality and ecological benefits

Vegetation biomass had significant positive relationships with SQI and EREI (*R* ^2^ > 0.6, *p*< 0.05), indicating that soil quality and ecological benefits improved with the increase of biomass in alpine grasslands. We found that the restoration measurements improved the soil quality and ecological benefits of desertified grassland (**Figures 2**, **3**), which was consistent with the findings of positive effects of the fenced enclosure on the soil quality of sandy grassland in the Tibetan Plateau as reported by Zhang and Zhao (2015); Hu et al. (2022), and Zhang et al. (2022). The main reason that was attributed to is that restored vegetation and/or sand barriers could be able to separate the sand sources and resist wind erosion effectively in areas with strong winds (Wan et al., 2023), which was beneficial for soil and water conservation as well as nutrient retention in desertified grassland. Moreover, plants grow preferably based on superior conditions of soil water and nutrients. On the one hand, vigorous plants could retain water and return organic matter *via* litterfall (Yu et al., 2018). On the other hand, the decomposition and humification of dead root and root exudates could promote soil quality (Wu et al., 2023). Therefore, the soil–vegetation system could form positive mutual feedback in restored grassland, further improving soil quality and restoring ecological service functions.

Soil quality and ecological benefits in ESSG were optimal among three restoration patterns because setting sand barriers was of great significance to prevent wind and fix sand as well as reserve moisture in sandy soils (Liu et al., 2019). Particularly, shrubs with deep roots promoted the accumulation of organic matter and nutrients in shallow soils combined with the roles of sand barriers (Kidron and Gutschick, 2013; Wang et al., 2021a), which could strengthen the positive mutual feedback between vegetation and soil. Similarly, Scotton (2019) also reported that combining sand fixation barriers and phytoremediation was a good way to restore the ecology of desertified grasslands.

Vegetation is an important and positive driving force for restoring desertified grasslands, as Wu et al. (2023) have reported. Furthermore, the significant improvement of vegetation (e.g., biomass) with the increase of restoration years was beneficial to restore ecological function directly and indirectly in desertified grasslands (Wang et al., 2023). As the restoration years of degraded grasslands in our study increased, the aboveground and root biomass of vegetation also gradually improved (**Table 1**). This was conductive to enhance the abilities of retaining water and nutrients and increase litter return and root exudates, which can also further strengthen the positive feedback between soil and vegetation. Thus, soil quality and ecological benefits increased significantly, corresponding to the increasing restoration years in each restoration pattern.

### 4.4 Optimal restoration year of applying management practices in different restored grasslands

The increasing rate of SQI in EG and ESG as well as EREI in EG began to decrease after 5 years of the application of restoration measurement, indicating that short-term fencing was more beneficial than long-term fencing. A probable explanation for this outcome was that the increasing requirement of plant nutrients slowed down the amount of nutrient accumulation and optimization of microbial characteristics in soils under the circumstances of long-term grazing exclusion (Li et al., 2023c). A similar result has been shown in the study of Sun et al. (2020) and Zhang et al. (2022), respectively, such that longer-term grazing exclusion had a little effect on vegetation growth without the construction of sand prevention belts. Interestingly, the increasing rate of SQI and EREI in ESSG was reduced after 7–9 years. This may be ascribed to the fact that the decay of sand barriers combined with a large amount of requirement of plant nutrients could also slow down the amelioration of soil features after 7–9 years based on the result of Liang et al. (2023). Thus, it was hard to ensure that the soil–vegetation system was constantly a virtuous cycle with the increase of restoration years.

In addition, plant species diversity appeared to decrease along with increased restoration years (**Table 1**) because the growth of some plants may have been inhibited during grassland restoration (Li et al., 2023c). This would cause a weakening in the stability of ecosystems, according to a research reported by Zhou et al. (2017). As a result, combined with the increasing rate of SQI, EREI, and plant diversity, we suggest that the appropriate time of anthropogenic monitoring and management measurements, such as applying organic fertilizer in soils and mowing return, should be reasonably considered at the beginning of 7 years in ESSG and 5 years in the other two patterns to maintain sustainable ecological benefits.

## 5 Conclusions

Using the MDS and comprehensive index method, our study assessed the soil quality and ecological benefits in restored grasslands. The MDS of soil quality comprised seven key indicators: silt, TN, C/N, TK, MBC, MBP, and fungi. The increase of vegetation coverage, density, and biomass resulted in such a way that the soil physicochemical and microbial properties as well as the soil quality and ecological benefits were superior in restored grasslands than in unrestored grasslands. Furthermore, the positive effects of soil quality and ecological restoration increased upon extending the restoration years of restored grasslands, and they generally ranked in the order as follows: EG< ESG< ESSG. The increasing rates of SQI decreased after 5 years in EG and ESG and 7 years in ESSG, while that of EREI in EG was lower than ESSG in each restoration year. Therefore, ESSG was the best restoration pattern of desertified grasslands, especially for moving sandy grasslands. Scientific anthropogenic monitoring and management measurements should be carried out at the beginning of 5 years in EG and ESG as well as 7 years in ESSG. We are supposed to pay more attention to the anthropogenic management practices to maintain sustainable ecological restoration effects of desertified grassland in future research—for instance, applying organic fertilizer combined with mowing return might be needed to further form a virtuous cycle of soil–vegetation system.

## Data availability statement

The original contributions presented in the study are included in the article/**Supplementary Material**. Further inquiries can be directed to the corresponding author.

## Author contributions

YiL: Data curation, Writing – original draft, Writing – review & editing. SH: Data curation, Visualization, Writing – review & editing. SL: Data curation, Writing – original draft. YP: Funding acquisition, Writing – review & editing. SZ: Conceptualization, Writing – review & editing. TL: Conceptualization, Data curation, Writing – review & editing. XX: Conceptualization, Validation, Writing – review & editing. YJ: Conceptualization, Visualization, Writing – review & editing. GW: Conceptualization, Writing – review & editing. DY: Funding acquisition, Writing – review & editing. YuL: Visualization, Writing – review & editing.

## References

[B1] Andrews S. S. Karlen D. L. Cambardella C. A. (2004). The soil management assessment framework: A quantitative soil quality evaluation method. Soil Sci. Soc. Am. J. 68 (6), 1945–1962. doi: 10.2136/sssaj2004.1945

[B2] Askari M. S. Holden N. M. (2014). Indices for quantitative evaluation of soil quality under grassland management. Geoderma 230, 131–142. doi: 10.1016/j.geoderma.2014.04.019

[B3] Biswas S. Hazra G. C. Purakayastha T. J. Saha N. Mitran T. Roy S. S.. (2017). Establishment of critical limits of indicators and indices of soil quality in rice-rice cropping systems under different soil orders. Geoderma 292, 34–48. doi: 10.1016/j.geoderma.2017.01.003

[B4] Brookes P. C. Landman A. Pruden G. Jenkinson D. S. (1985). Chloroform fumigation and the release of soil nitrogen: a rapid direct extraction method to measure microbial biomass nitrogen in soil. Soil Biol. Biochem. 17 (6), 837–842. doi: 10.1016/0038-0717(85)90144-0

[B5] Cai Y. Zhao M. J. Shi Y. X. Khan I. (2020). Assessing restoration benefit of grassland ecosystem incorporating preference heterogeneity empirical data from Inner Mongolia Autonomous Region. Ecol. Indic. 117, 106705. doi: 10.1016/j.ecolind.2020.106705 32834781 PMC7361097

[B6] Chen W. Westerhoff P. Leenheer J. A. Booksh K. (2003). Fluorescence excitation-emission matrix regional integration to quantify spectra for dissolved organic matter. Environ. Sci. Technol. 37 (24), 5701–5710. doi: 10.1021/es034354c 14717183

[B7] Culman S. W. Snapp S. S. Freeman M. A. Schipanski M. E. Beniston J. Lal R.. (2012). Permanganate oxidizable carbon reflects a processed soil fraction that is sensitive to management. Soil Sci. Soc. Am. J. 76 (2), 494–504. doi: 10.2136/sssaj2011.0286

[B8] Dong S. K. Wang X. X. Liu S. L. Li Y. Y. Su X. K. Wen L.. (2014). Reproductive responses of alpine plants to grassland degradation and artificial restoration in the Qinghai-Tibetan Plateau. Grass. Forage. Sci. 70 (2), 229–238. doi: 10.1111/gfs.12114

[B9] Frankeberger W. T. Johanson J. B. (1983). Method of measuring invertase activity in soils. Plant Soil. 74 (3), 301–311. doi: 10.1007/BF02181348

[B10] Guan S. Y. (1986). Soil Enzyme and its Research Method (Beijing, China: China agricultural science technology press), 274–314.

[B11] He Y. J. Han X. R. Wang X. P. Wang L. Q. Liang T. (2021). Long-term ecological effects of two artificial forests on soil properties and quality in the eastern Qinghai-Tibet Plateau. Sci. Total. Environ. 796, 148986. doi: 10.1016/j.scitotenv.2021.148986 34274659

[B12] Hou S. Xin M. X. Wang L. Jiang H. Li N. Wang Z. Q. (2014). The effects of erosion on the microbial populations and enzyme activity in black soil of northeastern China. Acta Ecol. Sin. 34 (6), 295–301. doi: 10.1016/j.chnaes.2014.10.001

[B13] Hu S. J. Hu R. Pu Y. L. (2018a). Influence of ecological restoration on soil biological fertility in Zoige desertification grassland. Pratacult. Sci. 35 (11), 2550–2560. doi: 10.11829/j.issn.1001-0629.2018-0069

[B14] Hu Z. M. Li S. G. Guo Q. Niu S. L. He N. P. Li L. H.. (2016b). A synthesis of the effect of grazing exclusion on carbon dynamics in grasslands in China. Glob. Chang. Biol. 22, 1385–1393. doi: 10.1111/gcb.13133 26485056

[B15] Hu R. Wang X. P. Zhang Y. F. Shi W. Jin Y. X. Chen N. (2016a). Insight into the influence of sand-stabilizing shrubs on soil enzyme activity in a temperate desert. Catena 137, 526–535. doi: 10.1016/j.catena.2015.10.022

[B16] Hu G. Y. Yu L. P. Dong Z. B. Lu J. F. Li J. Y. Wang Y. X.. (2018b). Holocene aeolian activity in the Zoige Basin, northeastern Tibetan plateau, China. Catena 160, 321–328. doi: 10.1016/j.catena.2017.10.005

[B17] Hu J. J. Zhou Q. P. Cao Q. H. Hu J. (2022). Effects of ecological restoration measures on vegetation and soil properties in semi-humid sandy land on the southeast Qinghai-Tibetan Plateau, China. Glob. Ecol. Conserv. 33, e02000. doi: 10.1016/j.gecco.2022.e02000

[B18] IUSS Working Group WRB. (2022). World reference base for soil resources. International soil classification system for naming soils and creating legends for soil maps. 4th edition (Vienna, Austria: International Union of Soil Sciences (IUSS)).

[B19] Jahany M. Rezapour S. (2020). Assessment of the quality indices of soils irrigated with treated wastewater in a calcareous semi-arid environment. Ecol. Indic. 109, 105800. doi: 10.1016/j.ecolind.2019.105800

[B20] Jin H. F. Zhong Y. J. Shi D. M. (2021). Quantifying the impact of tillage measures on the cultivated-layer soil quality in the red soil hilly region: Establishing the thresholds of the minimum data set. Ecol. Indic. 130, 108013. doi: 10.1016/j.ecolind.2021.108013

[B21] Kidron G. J. Gutschick V. P. (2013). Soil moisture correlates with shrub-grass association in the Chihuahuan Desert. Catena 107, 71–79. doi: 10.1016/j.catena.2013.02.001

[B22] Kooch Y. Amani M. Abedi M. (2022). The effect of shrublands degradation intensity on soil organic matter-associated properties in a semi-arid ecosystem. Sci. Total. Environ. 853, 158664. doi: 10.1016/j.scitotenv.2022.158664 36096213

[B23] Li C. S. Liang H. Y. Gao D. Y. Wang Y. B. Jin K. D. Liu J. N.. (2023a). Comparative study on the effects of soil quality improvement between urban spontaneous groundcover and lawn. Ecol. Indic. 148, 110056. doi: 10.1016/j.ecolind.2023.110056

[B24] Li Y. J. Ma J. W. Li Y. Q. Jia Q. M. Shen X. Y. Xia X. H. (2023b). Spatiotemporal variations in the soil quality of agricultural land and its drivers in China from 1980 to 2018. Sci. Total. Environ. 892, 164649. doi: 10.1016/j.scitotenv.2023.164649 37271389

[B25] Li W. L. Shang X. J. Yan H. P. Xu J. Liang T. A. Zhou H. K. (2023c). Impact of restoration measures on plant and soil characteristics in the degraded alpine grasslands of the Qinghai Tibetan Plateau: A meta-analysis. Agric. Ecosyst. Environ. 347, 108394. doi: 10.1016/j.agee.2023.108394

[B26] Liang Y. M. Gao Y. Meng Z. J. Han Y. L. Wang R. D. Duan X. T. (2023). Natural degradation process of Salix psammophila sand barriers regulates desert soil microbial biomass C: N: P stoichiometry and homeostasis. Catena 222, 106880. doi: 10.1016/j.catena.2022.106880

[B27] Liu X. X. Ding J. Y. Zhao W. W. (2023). Divergent responses of ecosystem services to afforestation and grassland restoration in the Tibetan Plateau. J. Environ. Manage. 344, 118471. doi: 10.1016/j.jenvman.2023.118471 37364488

[B28] Liu J. Wu L. C. Chen D. Li M. Wei C. J. (2017). Soil quality assessment of different Camellia oleifera stands in mid-subtropical China. Appl. Soil Ecol. 113, 29–35. doi: 10.1016/j.apsoil.2017.01.010

[B29] Liu L. Zeng K. Wu N. Zhang X. Q. Sun F. D. Chen D. M.. (2019). Variation in physicochemical and biochemical soil properties among different plant species treatments early in the restoration of a desertified alpine meadow. LAND. DEGRAD. Dev. 30 (16), 1889–1903. doi: 10.1002/ldr.3376

[B30] Liu M. Zhang Z. C. Sun J. Li Y. R. Liu Y. Berihun M. L.. (2020). Restoration efficiency of short-term grazing exclusion is the highest at the stage shifting from light to moderate degradation at Zoige, Tibetan Plateau. Ecol. Indic. 114, 106323. doi: 10.1016/j.ecolind.2020.106323

[B31] Lu R. K. (2000). Methods of soil chemical analysis (Beijing, China: China agricultural science technology press), 146–195.

[B32] Mamehpour N. Rezapour S. Ghaemian N. (2021). Quantitative assessment of soil quality indices for urban croplands in a calcareous semi-arid ecosystem. Geoderma 382, 114781. doi: 10.1016/j.geoderma.2020.114781

[B33] Mariano E. Jones D. L. Hill P. W. Trivelin P. C. O. (2016). Mineralisation and sorption of dissolved organic nitrogen compounds in litter and soil from sugarcane fields. Soil Biol. Biochem. 103, 522–532. doi: 10.1016/j.soilbio.2016.10.004

[B34] Martín-Sanz J. P. Santiago-Martín A. Valverde-Asenjo I. Quintana-Nieto J. R. Gonzalez-Huecas C. Lopez-Lafuente A. L. (2022). Comparison of soil quality indexes calculated by network and principal component analysis for carbonated soils under different uses. Ecol. Indic. 143, 109374. doi: 10.1016/j.ecolind.2022.109374

[B35] Nelson D. W. Sommers L. E. (1983). Total carbon, organic carbon, and organic matter. Methods Soil Analysis.: Part 2 Chem. Microbiol. Properties. 9, 539–579. doi: 10.2136/sssabookser5.3.c34

[B36] Raiesi F. (2017). A minimum data set and soil quality index to quantify the effect of land use conversion on soil quality and degradation in native rangelands of upland arid and semiarid regions. Ecol. Indic. 75, 307–320. doi: 10.1016/j.ecolind.2016.12.049

[B37] Romaniuk R. Giuffre L. Costantini A. Bartoloni N. Nannipieri P. (2011). A comparison of indexing methods to evaluate quality of soils: the role of soil microbiological properties. Soil Res. 49 (8), 733–741. doi: 10.1071/SR11147

[B38] Samborska A. Stepniewska Z. Stepniewski W. (2004). Influence of different oxidation states of chromium (VI, III) on soil urease activity. Geoderma 122 (2–4), 317–322. doi: 10.1016/j.geoderma.2004.01.019

[B39] Santos-Francés F. Martínez-Graña A. Ávila-Zarza C. Criado M. Sanchez Y. (2019). Comparison of methods for evaluating soil quality of semiarid ecosystem and evaluation of the effects of physico-chemical properties and factor soil erodibility (Northern Plateau, Spain). Geoderma 354, 113872. doi: 10.1016/j.geoderma.2019.07.030

[B40] Scotton M. (2019). Mountain grassland restoration: Effects of sowing rate, climate and soil on plant density and cover. Sci. Total. Environ. 651, 3090–3098. doi: 10.1016/j.scitotenv.2018.10.192 30463159

[B41] Shamiyeh N. B. Johnson L. F. (1973). Effect of heptachlor on numbers of bacteria, actinomycetes and fungi in soil. Soil Biol. Biochem. 5 (3), 309–314. doi: 10.1016/0038-0717(73)90078-3

[B42] Sun J. Liu M. Fu B. J. Kemp D. Zhao W. W. Liu G. H.. (2020). Reconsidering the efficiency of grazing exclusion using fences on the Tibetan Plateau. Sci. Bull. 65 (16), 1405–1414. doi: 10.1016/j.scib.2020.04.035 36659220

[B43] Tabatabai M. (1994). Methods of soil analyses, part 2, microbiological and biochemical properties (Madison, WI, USA: Soil Science Society of America).

[B44] Vance E. D. Brookes P. C. Jenkinson D. S. (1987). An extraction method for measuring soil microbial biomass C. Soil Biol. Biochem. 19 (6), 703–707. doi: 10.1016/0038-0717(87)90052-6

[B45] Wan R. P. Luo D. Y. Liu J. Y. Zhang Y. Xiang Y. Q. Yan W.. (2023). Superior improvement on soil quality by Pennisetum sinese vegetation restoration in the dry-hot valley region, SW China. Sci. Total. Environ. 878, 163185. doi: 10.1016/j.scitotenv.2023.163185 37004763

[B46] Wang W. L. Dong Z. B. Yan C. Z. (2014). Trend analysis on land degradation in Zoige Plateau based on landscape structure methods. J. ARID. LAND. 28 (10), 117–122. doi: 10.13448/j.cnki.jalre.2014.10.020

[B47] Wang R. D. Gao Y. Dang X. H. Yang X. Liang Y. M. Zhao C. (2021a). Microstructure and biodegradation of long-established Salix psammophila sand barriers on sand dunes. Environ. Technol. Innov. 21, 101366. doi: 10.1016/j.eti.2021.101366

[B48] Wang T. Xue X. Zhou L. Guo J. (2012). Combating aeolian desertification in northern China. Land. Degrad. Dev. 26, 118–132. doi: 10.1002/ldr.2190

[B49] Wang J. Zhao W. W. Wang G. Yang S. Q. Pereira P. (2021b). Effects of long-term afforestation and natural grassland recovery on soil properties and quality in Loess Plateau (China). Sci. Total. Environ. 770, 144833. doi: 10.1016/j.scitotenv.2020.144833 33508670

[B50] Wang J. Zhao W. W. Xu Z. X. Ding J. Y. Yan Y. Ferreira C. S. S. (2023). Plant functional traits explain long-term differences in ecosystem services between artificial forests and natural grasslands. J. Environ. Manage. 345, 118853. doi: 10.1016/j.jenvman.2023.118853 37660423

[B51] Weber J. M. Tihanyi K. (1994). Methods in enzymology, [41] adenovirus endopeptidases. Acad. Press. 244, 595–604. doi: 10.1016/0076-6879(94)44043-3 7845235

[B52] Wu W. J. Chen G. J. Meng T. F. Li C. Feng H. Si B. C.. (2023). Effect of different vegetation restoration on soil properties in the semi-arid Loess Plateau of China. Catena 220, 106630. doi: 10.1016/j.catena.2022.106630

[B53] Wu C. S. Liu G. H. Huang C. Liu Q. S. (2019). Soil quality assessment in Yellow River Delta: Establishing a minimum data set and fuzzy logic model. Geoderma 334, 82–89. doi: 10.1016/j.geoderma.2018.07.045

[B54] Wu P. F. Zhang H. Z. Wang Y. (2015). The response of soil macroinvertebrates to alpine meadow degradation in the Qinghai-Tibetan Plateau, China. Appl. Soil Ecol. 90, 60–67. doi: 10.1016/j.apsoil.2015.02.006

[B55] Wu Z. G. Zhu D. Y. Xiong K. N. Wang X. F. (2022). Dynamics of landscape ecological quality based on benefit evaluation coupled with the rocky desertification control in South China Karst. Ecol. Indicators. 138, 108870. doi: 10.1016/j.ecolind.2022.108870

[B56] Yang T. H. Li X. J. Hu B. Wei D. D. Wang Z. L. Bao W. K. (2022). Soil microbial biomass and community composition along a latitudinal gradient in the arid valleys of southwest China. Geoderma 413, 115750. doi: 10.1016/j.geoderma.2022.115750

[B57] Yang Z. A. Su Q. Q. Chen H. Yang G. (2021). Anthropogenic impacts recorded by a 200-year peat profile from the Zoige Peatland, northeastern Qinghai-Tibetan Plateau. Catena 206, 105463. doi: 10.1016/j.catena.2021.105463

[B58] Yao R. J. Yang J. S. Gao P. Zhang J. B. Jin W. H. (2013). Determining minimum data set for soil quality assessment of typical salt-affected farmland in the coastal reclamation area. Soil Till. Res. 128, 137–148. doi: 10.1016/j.still.2012.11.007

[B59] Yu P. J. Han D. L. Liu S. W. Wen X. Huang Y. X. Jia H. T. (2018). Soil quality assessment under different land uses in an alpine grassland. Catena 171, 280–287. doi: 10.1016/j.catena.2018.07.021

[B60] Zhang Z. W. Han J. H. Yin H. Y. Xue J. Jia L. Z. Zhen X.. (2022). Assessing the effects of different long-term ecological engineering enclosures on soil quality in an alpine desert grassland area. Ecol. Indic. 143, 109426. doi: 10.1016/j.ecolind.2022.109426

[B61] Zhang Q. Liu K. S. Shao X. Q. Li H. He Y. X. Wang B. J.. (2021). Microbes require a relatively long time to recover in natural succession restoration of degraded grassland ecosystems. Ecol. Indic. 129, 107881. doi: 10.1016/j.ecolind.2021.107881

[B62] Zhang C. Liu G. B. Xue S. Song Z. L. (2011). Rhizosphere soil microbial activity under different vegetation types on the Loess Plateau, China. Geoderma 161 (3–4), 115–125. doi: 10.1016/j.geoderma.2010.12.003

[B63] Zhang Y. Y. Zhao W. Z. (2015). Vegetation and soil property response of short-time fencing in temperate desert of the Hexi Corridor, northwestern China. Catena 133, 43–51. doi: 10.1016/j.catena.2015.04.019

[B64] Zhou Y. Ma H. B. Xie Y. Z. Jia X. Y. Su T. T. Li J. P.. (2020). Assessment of soil quality indexes for different land use types in typical steppe in the loess hilly area, China. Ecol. Indic. 118, 106743. doi: 10.1016/j.ecolind.2020.106743

[B65] Zhou Z. H. Wang C. K. Jin Y. (2017). Stoichiometric responses of soil microflora to nutrient additions for two temperate forest soils. Biol. Fertil. Soils 53 (4), 397–406. doi: 10.1007/s00374-017-1188-y

