# Supplementary material for: Soil quality and ecological benefits assessment of alpine desertified grassland following different ecological restoration measures
Source: Front Plant Sci. 2023 Oct 25;14:1283457. doi: 10.3389/fpls.2023.1283457 (PMC10634470; doi:10.3389/fpls.2023.1283457)
Supplement: Supplementary file 1 [file DataSheet_1.docx]

**Supplementary Material**

Graphical Abstract

Table S1 Analysis of one-way variance of soil quality indicators for unrestored grassland.

Table S2 Analysis of one-way variance among depths of soil quality indicators.

Table S3 Correlation coefficients of soil chemical indicators.

Table S4 Correlation coefficients of soil microbial indicators.

Table S5 Microbial properties among different restoration measurements.

Fig. S1 Relationships among microorganisms and soil physicochemical indicators.

Table S1 Analysis of one-way variance of soil quality indicators for unrestored grassland.

| Soil indicator | *F* Value | *P* Value |  | Soil indicator | *F* Value | *P* Value |
| --- | --- | --- | --- | --- | --- | --- |
| BD | 1.048 | 0.394 |  | AK | 4.934 | 0.140 |
| Sand | 2.800 | 0.120 |  | Actinomyces | 4.224 | 0.074 |
| Silt | 2.725 | 0.125 |  | Fungi | 1.404 | 0.300 |
| Clay | 0.267 | 0.620 |  | MBC | 3.092 | 0.117 |
| TOC | 1.513 | 0.277 |  | MBN | 3.473 | 0.071 |
| TN | 3.823 | 0.068 |  | MBP | 3.732 | 0.072 |
| DON | 3.929 | 0.065 |  | Urease | 3.712 | 0.090 |
| NH_4_^+^-N | 1.471 | 0.286 |  | Protease | 2.372 | 0.116 |
| NO_3_^-^-N | 0.849 | 0.463 |  | Sucrase | 3.318 | 0.102 |
| TP | 0.286 | 0.759 |  | Amylase | 2.020 | 0.081 |
| AP | 3.860 | 0.103 |  | Neutral phosphatase | 2.617 | 0.216 |
| TK | 4.500 | 0.149 |  | Alkaline phosphatase | 1.177 | 0.310 |

Abbreviations: BD, Bulk density; TOC, Total organic carbon; TN, Total nitrogen; DON, Dissolved organic nitrogen; NH_4_^+^-N, Ammonium nitrogen; NO_3_^-^-N, Nitrate nitrogen; TP, Total phosphorus; AP, Available phosphorus; TK, Total potassium; AK, Available potassium; MBC, Microbial biomass carbon; MBN, Microbial biomass nitrogen; MBP, Microbial biomass phosphorus.

Table S2 Analysis of one-way variance among depths of soil indicators.

| Soil indicator | *F* Value | *P* Value |  | Soil indicator | *F* Value | *P* Value |
| --- | --- | --- | --- | --- | --- | --- |
| BD | 1.661 | 0.195 |  | AK | 2.362 | 0.099 |
| Sand | 0.087 | 0.917 |  | Actinomyces | 1.867 | 0.159 |
| Silt | 0.265 | 0.767 |  | Fungi | 2.340 | 0.101 |
| Clay | 0.020 | 0.980 |  | MBC | 0.819 | 0.444 |
| TOC | 1.115 | 0.332 |  | MBN | 0.627 | 0.536 |
| TN | 0.897 | 0.411 |  | MBP | 0.950 | 0.390 |
| DON | 2.594 | 0.079 |  | Urease | 0.261 | 0.771 |
| NH_4_^+^-N | 0.387 | 0.680 |  | Protease | 1.299 | 0.277 |
| NO_3_^-^-N | 0.107 | 0.898 |  | Sucrase | 1.491 | 0.230 |
| TP | 0.069 | 0.933 |  | Amylase | 0.450 | 0.639 |
| AP | 0.243 | 0.785 |  | Neutral phosphatase | 1.034 | 0.359 |
| TK | 0.740 | 0.479 |  | Alkaline phosphatase | 3.257 | 0.042 |

Abbreviations: BD, Bulk density; TOC, Total organic carbon; TN, Total nitrogen; DON, Dissolved organic nitrogen; NH_4_^+^-N, Ammonium nitrogen; NO_3_^-^-N, Nitrate nitrogen; TP, Total phosphorus; AP, Available phosphorus; TK, Total potassium; AK, Available potassium; MBC, Microbial biomass carbon; MBN, Microbial biomass nitrogen; MBP, Microbial biomass phosphorus.

Table S3 Correlation coefficients of soil chemical indicators.

| Soil indicators | TOC | TN | NH_4_^+^-N | NO_3_^-^-N | TP | AP |
| --- | --- | --- | --- | --- | --- | --- |
| TOC | 1 |  |  |  |  |  |
| TN | 0.871^**^ | 1 |  |  |  |  |
| NH_4_^+^-N | 0.802^**^ | 0.876^**^ | 1 |  |  |  |
| NO_3_^-^-N | 0.699^**^ | 0.782^**^ | 0.838^**^ | 1 |  |  |
| TP | 0.697^**^ | 0.711^**^ | 0.702^**^ | 0.751^**^ | 1 |  |
| AP | 0.776^**^ | 0.775^**^ | 0.737^**^ | 0.696^**^ | 0.743^**^ | 1 |
| Sum of correlation coefficients | 4.845 | 5.016 | 4.956 | 4.766 | 4.604 | 4.728 |

**: Correlation is significant at *p* < 0.01. Abbreviations: TOC, Total organic carbon; TN, Total nitrogen; NH_4_^+^-N, Ammonium nitrogen; NO_3_^-^-N, Nitrate nitrogen; TP, Total phosphorus; AP, Available phosphorus.

Table S4 Correlation coefficients of soil microbial indicators.

| Soil indicators | Bacteria | MBC | MBN | Urease | Protease | Sucrase | Amylase |
| --- | --- | --- | --- | --- | --- | --- | --- |
| Bacteria | 1 |  |  |  |  |  |  |
| MBC | 0.882^**^ | 1 |  |  |  |  |  |
| MBN | 0.860^**^ | 0.936^**^ | 1 |  |  |  |  |
| Urease | 0.850^**^ | 0.902^**^ | 0.903^**^ | 1 |  |  |  |
| Protease | 0.911^**^ | 0.874^**^ | 0.868^**^ | 0.855^**^ | 1 |  |  |
| Sucrase | 0.756^**^ | 0.791^**^ | 0.792^**^ | 0.768^**^ | 0.722^**^ | 1 |  |
| Amylase | 0.806^**^ | 0.827^**^ | 0.833^**^ | 0.811^**^ | 0.802^**^ | 0.838^**^ | 1 |
| Sum of correlation coefficients | 6.067 | 6.212 | 6.192 | 6.090 | 6.032 | 5.668 | 5.917 |

**: Correlation is significant at *p* < 0.01. Abbreviations: MBC, Microbial biomass carbon; MBN, Microbial biomass nitrogen.

Table S5 Microbial properties among different restoration measurements.

| Indicators | CK |  | EG | | |  | ESG | | |  | ESSG | | |
| --- | --- | --- | --- | --- | --- | --- | --- | --- | --- | --- | --- | --- | --- |
|  |  |  | ≤ 5 years | 7–9 years | ≥ 15 years |  | ≤ 5 years | 7–9 years | ≥ 15 years |  | ≤ 5 years | 7–9 years | ≥ 15 years |
| MBC (mg kg^-1^) | 40.28a |  | 69.98Ac | 104.08Ab | 148.43Ba |  | 69.68Ac | 88.84Ab | 180.72Aa |  | 46.95Bc | 101.52Ab | 177.19Aa |
| MBN (mg kg^-1^) | 2.74a |  | 6.95ABb | 10.62Ab | 15.62Aa |  | 8.47Ab | 9.82Ab | 17.64Aa |  | 5.48Ba | 10.07Ab | 18.43Aa |
| MBP (mg kg^-1^) | 0.88a |  | 1.24Bb | 1.67Bb | 3.00Aa |  | 1.27Bb | 1.75Bb | 2.51Aa |  | 2.29Ab | 2.74Ab | 4.01Aa |
| Urease (ug g^-1^ h^-1^) | 2.37a |  | 6.88Ab | 8.82Ab | 14.11Ba |  | 4.49Bc | 8.35Ab | 16.35Aa |  | 6.96Ac | 10.20Ab | 16.04Aa |
| Sucrase (ug g^-1^ h^-1^) | 77.21a |  | 741.71Ab | 1001.61Aab | 1270.14Aa |  | 764.96Ab | 898.06Aab | 1071.92Ba |  | 436.06Bb | 1077.20Aa | 1202.04ABa |
| Amylase (ug g^-1^ h^-1^) | 3.66a |  | 8.55Ab | 14.62Aab | 19.51Ba |  | 10.59Aa | 13.05Aa | 14.04Ca |  | 7.96Ac | 17.22Ab | 24.97Aa |
| Protease (ug g^-1^ h^-1^) | 11.82a |  | 23.97Ab | 40.95Aab | 57.74Ba |  | 23.59Ab | 29.43Ab | 65.61Ba |  | 25.53Ab | 31.27Ab | 82.16Aa |
| Neutral phosphatase (ug g^-1^ h^-1^) | 21.58a |  | 53.03Aa | 56.38Aa | 64.41Ba |  | 41.47Bb | 38.73Bb | 56.03Ba |  | 44.79ABc | 55.95Ab | 79.01Aa |
| Alkaline phosphatase (ug g^-1^ h^-1^) | 17.37a |  | 58.05ABb | 65.32Aab | 78.64Aa |  | 65.85Ab | 72.27Aab | 76.18Aa |  | 50.33Bb | 63.03Ab | 81.37Aa |
| Bacteria (10^6^ CFU g^-1^) | 4.28a |  | 8.30Ab | 12.80Ab | 18.21Ba |  | 9.69Ab | 7.94Ab | 20.11Ba |  | 8.82Ac | 11.99Ab | 23.71a |
| Fungi (10^3^ CFU g^-1^) | 2.50a |  | 5.65Ab | 7.47Ab | 10.34Aa |  | 5.27Ab | 4.52Ab | 8.67Aa |  | 3.02Bb | 5.65Aa | 4.78Bab |
| Actinomycetes (10^6^ CFU g^-1^) | 3.62a |  | 4.49Aa | 4.56Aa | 4.63Aa |  | 4.57Aa | 4.42Aa | 6.14Aa |  | 2.79Bc | 4.51Ab | 5.64Aa |

Abbreviations: MBC, Microbial biomass carbon; MBN, Microbial biomass nitrogen; MBP, Microbial biomass phosphorus. Different capital letters in the same row indicate significant differences at *p* < 0.05 among different restoration patterns. Different lowercase letters in the same row indicate significant differences at *p* < 0.05 among different restoration years. The data of soil microbial properties were organized from Hu et al. (2018a).

Fig. S1 Relationships among microorganisms and soil physicochemical indicators. Abbreviations: C/N, Carbon-nitrogen ratio; TN, Total nitrogen; TK, Total potassium; MBC, Microbial biomass carbon; MBP, Microbial biomass phosphorus.
